# A Mixed Methods Process Evaluation of a Food Hygiene Intervention in Low-Income Informal Neighbourhoods of Kisumu, Kenya

**DOI:** 10.1007/s10995-022-03548-6

**Published:** 2022-11-09

**Authors:** Sheillah Simiyu, Evalyne Aseyo, John Anderson, Oliver Cumming, Kelly K. Baker, Robert Dreibelbis, Jane Awiti Odhiambo Mumma

**Affiliations:** 1 African Population and Health Research Center, Manga Close, Off Kirawa Road, P.O Box 10787- 00100, Nairobi, Kenya; 2 Great Lakes University of Kisumu, P.O Box 2224-40100, Kisumu, Kenya; 3 Independent Research Consultant, 78702 Austin, TX USA; 4 Department of Disease Control, London School of Hygiene and Tropical Medicine, WC1E 7HT London, UK; 5 Department of Occupational and Environmental Health College of Public Health, University of Iowa, 52333 Iowa City, IA USA

**Keywords:** Diarrhoea, food hygiene, handwashing with soap, low-income settlements, Process Evaluation

## Abstract

**Objectives:**

Diarrhoea is a leading cause of infant mortality with the main transmission pathways being unsafe water and contaminated food, surfaces and hands. The ‘Safe Start’ trial evaluated a food hygiene intervention implemented in a peri-urban settlement of Kisumu, Kenya, with the aim of reducing diarrhoeagenic enteric infections among infants. Four food hygiene behaviours were targeted: handwashing with soap before preparation and feeding, boiling infant food before feeding, storing infant food in sealed containers, and exclusive use of designated utensils during feeding.

**Methods:**

A process evaluation of the intervention was guided by a theory of change describing the hypothesised implementation and receipt of the intervention, mechanisms of change, and the context. These were assessed by qualitative and quantitative data that included debriefing sessions with the delivery teams and Community Health Volunteers (CHVs), and structured observations during food preparation.

**Results:**

The intervention achieved high coverage and fidelity with over 90% of 814 eligible caregivers participating in the intervention. Caregivers in the intervention arm demonstrated an understanding of the intervention messages, and had 1.38 (95% CI: 1.02–1.87) times the odds of washing hands before food preparation and 3.5 (95% CI: 1.91–6.56) times the odds of using a feeding utensil compared to caregivers in the control group. Contextual factors, especially the movement of caregivers within and outside the study area and time constraints faced by caregivers influenced uptake of some intervention behaviours.

**Conclusion:**

Future interventions should seek to explicitly target contextual factors such as secondary caregivers and promote food hygiene interventions as independent of each other.

## Significance

What is already known on this subject.

Diarrhoea is known to be a significant cause of infant morbidity and mortality, mainly through unsafe water and food, and mainly from rural areas.

What this study adds.

This study describes the implementation of a novel food hygiene intervention to reduce diarrhoea causing enteric infections among children in low income peri urban settlements. The manuscript details the delivery of the intervention, describes the context, and explores associations influencing adoption of the intervention. Findings of the evaluation are meant to support the results of the trial.

## Background

Diarrhoea is among the top five leading causes of years of life lost, and the eighth leading cause of mortality in low-income countries; causing an estimated 1.6 million deaths, a quarter of which are among children under five years (Naghavi et al., 2017; Troeger et al., 2020). Childhood diarrhoea is further associated with growth faltering due to an increased risk of infectious disease (Troeger et al., 2018). Diarrhoeal diseases are generally transmitted faecal-orally by various environmental pathways including unsafe water and food, and contaminated surfaces and hands. Food in particular is estimated to account for a third of all diarrhoeal diseases (Kirk et al., 2017; Hald et al., 2016).

High levels of infant and child food contamination have been reported in high-burden settings, especially after the introduction of weaning foods (Parvez et al., 2017; Tsai et al., 2019; Bick et al., 2020; Islam et al., 2013), and there have been a number of recent studies to assess interventions to reduce child food contamination (Islam et al., 2013; Touré et al., 2013) or to improve food hygiene behaviours among caregivers (Gautam et al., 2017; Manaseki-Holland et al., 2021). Most of these studies were conducted in rural areas/areas outside the towns (Islam et al., 2013; Gautam et al., 2017; Manaseki-Holland et al., 2021) and examined infant food contamination by testing for the presence or absence of faecal indicator bacteria. Studies in low income peri-urban areas (areas transitioning from rural land use to urban centres, or in the periphery of urban and rural areas) that describe food contamination from a range of diarrhoea causing pathogens are few (Touré et al., 2013).

The ‘Safe Start’ trial (clinical trials ref: NCT03468114) evaluated an intervention to reduce diarrhoeagenic enteric infections among infants in low-income neighbourhoods (Mumma, 2019). The trial was delivered in a low income peri-urban setting, and it used a novel measure of stool-based enteric pathogen detection as a health outcome, along with caregiver reported diarrhoea (Mumma, 2019). In this manuscript, we detail and evaluate the implementation process of the ‘Safe Start’ intervention. The aim of the process evaluation was to document the delivery of the intervention (implementation), provide an understanding of the context in which the intervention was delivered (context), and explore associations between exposure to and adoption of the intervention (receipt and mechanisms of change). Findings from this process evaluation will be used to support the interpretation of trial results, identify elements of the intervention that were effective or ineffective, and strengthen future intervention designs (Linnan & Steckler, 2002; World Health Organization, 2000; Moore et al., 2015).

## Trial and Study Setting

The trial was implemented between March 2018 and June 2019 in the neighbourhoods of Nyalenda A and B, both of which are low-income, peri-urban settlements of Kisumu. Located in western Kenya in Kisumu County, Kisumu city is the third largest city in Kenya, with a population of approximately 500,000 (Kenya National Bureau of Statistics, 2019). Diarrhoeal diseases are the third leading cause of morbidity among children under the age of five in Kisumu County (County Government of Kisumu, 2018). Approximately 60% of the population in Kisumu city lives in low-income settlements characterized by poverty, inadequate water and sanitation, and poor housing (National Council for Population and Development, 2013). Our prior and trial-related research confirmed that supplemental foods are a source of faecal indicator and enteric pathogen exposure and that inadequate household food hygiene is a leading source of contamination (Hoffmann et al., 2020). Residents in the settlements, including those in Nyalenda have a mix of urban and rural lifestyle, with landowners constructing rental housing units to meet the growing demand for housing.

Trial participants were infants aged between 22 and 37 weeks and their caregivers. A primary caregiver was defined as an individual who was directly responsible for the infant while a secondary caregiver was any other individual who supported the primary caregiver in watching over the infant. The intervention targeted four food hygiene behaviours: handwashing with soap before food preparation and infant feeding, boiling infant food before feeding, storing infant food in sealed containers, and feeding the infant using feeding utensils that have been designated for the infant and reserved from other use (Mumma, 2019). Development of the intervention followed the Behaviour Centred Design (BCD) approach (Aunger & Curtis, 2016). The intervention consisted of a mixture of items given to primary caregivers to prompt and enable targeted behaviours combined with motivational and educational messaging focusing on food hygiene and the “nurture” motive. Intervention components were assessed and refined iteratively following Trial of Improved Practice (TIPs) methodology (Simiyu et al., 2020). Full details of the intervention, planned implementation, and trial design have been described in detail (Mumma, 2019; Simiyu et al., 2020).

The trial had a planned sample size of 750 infants (Mumma, 2019). Infants were enrolled if they were 22 weeks (+/- 1 week), and if their caregivers were resident in the study area and were planning to reside in the study area for the subsequent 5 months. Infants who met this eligibility criteria were enrolled at 22 weeks of age (5.5 months) and a baseline survey and stool sample collected. The intervention was then delivered in four visits when the infants were 23, 25, 29 and 32 weeks of age to coincide with ages of infants before 6 months, at 6 months, at 7 months, and at 8 months respectively (Mumma, 2019) A follow-up midline survey was completed and a food sample collected when the infants were 33 weeks (8.2 months), and a final endline survey and stool sample collected when the infants were 37 weeks (9.2 months) (Mumma, 2019). The surveys and sample collection were carried out by a team of enumerators (trained specifically for research activities) while the intervention visits were carried out by another team of enumerators (trained specifically for intervention activities) in collaboration with Community Health Volunteers (CHVs). The full protocol of the ‘Safe Start’ trial has been published (Mumma, 2019).

## Pre-intervention Activities

### CHV Preparation Activities

A total of 54 CHVs were randomly allocated in equal number to control and intervention arms. CHVs in the intervention arm were trained on the study goals and objectives, food hygiene messages to be delivered to caregivers during the visits, capturing information of their visits on tracking tools, and working with the trained intervention team. In addition to visiting the caregivers when the infants were 23 and 29 weeks, the CHVs in the intervention arm accompanied the intervention teams during the second and fourth intervention visits when the infants were 25 and 32 weeks old, respectively. CHVs in the control arm were trained on the overall aspects of the study, the importance of making the bimonthly household visits according to scheduled guidelines from the Ministry of Health (MoH), capturing health situation information as required on MoH forms, and responding to any concerns by caregivers.

### Research Activities Training

A team of field staff (enumerators) who were involved in research activities collected data at baseline, midline, and end line visits. This team was trained on various aspects of the trial, including consent procedures, procedures in handling and transportation of food and stool samples, and survey questionnaire administration.

### Intervention Activities Training

Field staff who were involved in delivery of the intervention were assigned to the intervention arm and the control arm. The field staff assigned to the intervention arm were trained on engaging the caregivers during the visits by explaining the importance of improved food hygiene behaviours and the need to track diarrhoea, capturing information from the caregivers by audio recording and taking notes, and responding to any questions and concerns from caregivers. They were provided with scripts and activities that were completed with each household, which were designed to target the specific motives of nurture. The field staff assigned to the control arm were also trained on engaging caregivers, explaining the need to track diarrhoea, and answering any questions from the caregivers. Both teams in the intervention and control arms visited the caregivers when the infants were 25 and 32 weeks old. Care was taken so that the team of field staff involved in intervention activities and the team involved in research activities did not cross paths during the data collection or intervention delivery activities.

## Methods

**Fig. 1:**
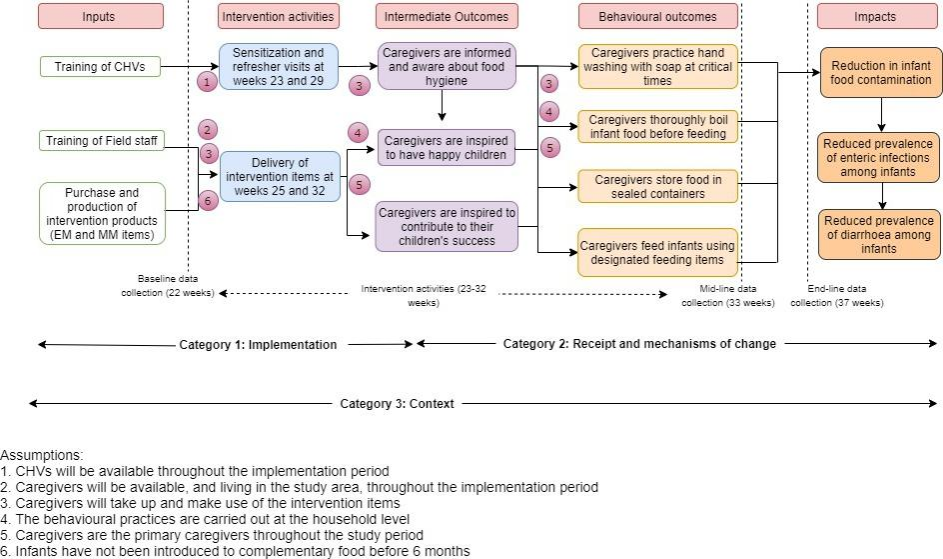
Theory of Change of the ‘Safe Start’ intervention

The Theory of Change (ToC) for the intervention that was used to guide the process evaluation is depicted in Fig. 1. The ToC shows how the intervention was hypothesized to impact upon infant health.

Our process evaluation builds on published methods and guidance (Linnan & Steckler, 2002; World Health Organization, 2000; Moore et al., 2015; Grant et al., 2013) and directly assessed three broad categories and eight specific intervention components. These categories and components as related to the ‘Safe Start’ intervention are defined in Table 1.

**Table 1:** Process evaluation categories and components of the Safe Start intervention

Category	Component	Definition
Implementation	Reach	The proportion of caregivers who came into contact with/ participated in the intervention
	Dose	The number of visits made to each of the intervention households
	Fidelity	The extent to which the intervention was delivered as conceived
	Recruitment	Procedures used to enrol participants in the intervention
Receipt and change mechanisms	Participant engagement	Caregivers’ understanding of the intervention messages
	Participant responses	Caregivers interaction with key messages
	Acceptance	Utilisation and behavioural uptake of the intervention by caregivers
Context	Context	Factors in the local environment (social, political, economic, cultural) that act as barriers or facilitators to intervention delivery and uptake.

### Data Collection

Data that supported the process evaluation were collected using qualitative and quantitative methods. An overview of data collection methods, the information sought using these methods, and timelines when the data were collected is provided in Table 2.

**Table 2:** Process evaluation methods of the ‘Safe Start’ intervention

Days since enrolment^¥^	Target Infant age	Activity	Data collection methods	Respondents	Information sought
0	22 weeks	Enrolment	Baseline survey, Stool collection	Caregivers	Age of infants, household information, willingness to participate in the trial, collection of infants stool
7	23 weeks	Visit 1: Sensitisation	CHV records, Debriefing sessions with CHVs	CHVs	Visits made to caregivers, Information relayed, challenges faced during the visits
21	25 weeks	Visit 2: Intervention delivery	Debriefing sessions with intervention teams	Intervention and control teams	Number of visits made, Information relayed to caregivers, challenges mentioned by caregivers, other behavioural practices mentioned by caregivers
49	29 weeks	Visit 3: Refresher	CHV records, Debriefing sessions with CHVs	CHVs	Visits made to caregivers, Information relayed, challenges faced during the visits
70	32 weeks	Visit 4: Intervention delivery	Debriefing sessions with intervention teams	Intervention and control teams	Number of visits made to the caregivers, uptake of intervention items
77	33 weeks	Midline visit	Midline survey, Structured observation	Caregivers	Completed visits, caregivers’ behavioural practices

^¥^The visits could be done within a window of ± 7 days

- Household surveys.

Household surveys were conducted at three time points based on the age of the enrolled infant: baseline (22 weeks), midline (33 weeks) and end line (37 weeks). The surveys included a short questionnaire administered to the infant’s caregiver and captured information on household characteristics, access to water, handwashing with soap, household sanitation, infant breastfeeding and supplemental feeding practices, and infant health. At the baseline and end line visits, the field staff involved in research activities collected stool samples from the infants which were taken to the laboratory for analysis. Households who provided a stool sample were enrolled into the intervention.

b. Debriefing sessions with the team involved in intervention activities.

During the fourth visits to households, the intervention field staff discussed food hygiene with household members, as well as caregivers’ experiences in using the intervention items and tracking of diarrhoea. These discussions with households were audio recorded. The study team conducted routine debriefing sessions with the intervention field staff to discuss intervention delivery, challenges encountered by the field staff and the participating households, and deviations from planned delivery. During these debriefing sessions, household calendars were also collected. Quantitative data on the number of visits made by the intervention field staff provided information on reach, dose and fidelity; while the recorded qualitative data from the discussions with caregivers provided rich information on the caregivers’ engagement, response and acceptance, as well as an understanding of the context.

c. Structured Observations.

During the midline survey, the field staff involved in research activities completed structured observations on the caregivers’ practices on handwashing, infant food preparation, feeding, storage and the use and/or display of the intervention materials Mumma, 2019). The structured observation data were used to assess reach, dose, and acceptance of the intervention.

d. CHV Records and debriefing sessions with CHVs.

CHVs were provided with detailed forms to complete at each household visit. The forms required the CHVs to record the caregiver’s name, location, date of the visit, topics that were discussed during the visits, and whether the caregivers received the intervention items. The caregivers signed on the forms to confirm the visit and the topics discussed by the CHV. The study team held monthly debriefing sessions with CHVs and discussed lessons learnt during the monthly visits, the number of households not visited, and the reasons why caregivers had not been visited. The forms were also retrieved during these meetings. Quantitative data from these forms provided information on fidelity and dose, while qualitative data from the discussions complemented the quantitative data and provided in depth information about the context.

### Ethical Requirements

#### Ethical Approvals

 for the study were obtained from Great Lakes University of Kisumu (Ref: GREC/010/248/2016), the London School of Hygiene and Tropical Medicine (Ref: 14695), and from the University of Iowa (Ref: 201804204). A participant information sheet (PIS) and consent statement were read to the participating caregivers in their preferred language (English, Swahili or Luo). Those wishing to participate consented by signing the consent form and they were given copies for their safe keeping. Participants were allowed to withdraw from the study at any time.

### Data Management and Analysis

Quantitative data from the baseline, midline and end line was collected through the ODK platform on mobile tablets. The data was sent to a central server whose access was limited to the study management team. The data were cleaned and checked for inconsistencies, duplicates, and out of range values. Any inconsistent data was cross-checked to verify the anomaly. Descriptive statistics were performed to summarize the results. Two proportion Z-test was used to assess the distribution of participants across the groups during the intervention visits (i.e. to test the hypothesis of equal distribution/retention of participants in the control and intervention arms during the intervention visits). Statistical analyses considered the cluster randomisation through random effects logistic regression models, which were performed to compare the key food hygiene practices (outcomes) in the control and intervention groups and to test the hypothesis that the intervention led to improved food hygiene behaviours in the intervention group. Analysis were conducted at 95% CI and performed using STATA version 15 (Stata Corp, College Station, TX, USA) software.

For qualitative data, each of the intervention field staff transcribed their audio recordings verbatim into Microsoft Word and translated them back to English. The translated transcripts were then distributed to different members of the intervention field staff team to verify that the audio recordings were all transcribed and that the meanings were not lost during translation. During translation, transcripts were first subjected to multiple rounds of review in order to understand the storyline. During the first review, initial codes (such as recall of intervention items, uptake of behaviours, and use of intervention items) were identified and recorded, and they formed the initial codebook that guided the analysis.

All the transcripts were then transferred to ATLAS.ti (version 7) software and coded based on this initial codebook. The codebook was improved during the subsequent review and coding process with new codes, and the transcripts were re-read again to ensure that the additional codes had been captured. The codes were merged into ‘families’ and later categorised under the evaluation components of context, acceptance, and engagement. During analysis and presentation, qualitative data was used to explain the quantitative data. The combination of qualitative and quantitative data enriched the presentation and interpretation of the results.

### Reflexivity

The field staff who were involved in intervention activities were drawn from the community where the intervention was implemented, and this facilitated a rapport between the field team and the intervention participants. The main researchers had little interaction with the intervention participants, which enabled us to minimise bias in the analysis of the results. Discussions during the debriefing sessions enhanced self-awareness among the main researchers and the field staff involved in intervention activities, and we were made aware of the caregivers’ challenges and practices that we had not anticipated; for example, we understood that dwellers in low-income settlements are not permanent dwellers, and they often move to other areas or travel to the village. This awareness was beneficial during analysis as it enabled a constructive interpretation of the context and behavioural practices of the caregivers.

## Results

The results are presented according to the two categories of implementation and receipt and mechanisms of change. Contextual factors - aspects in the local environment that were barriers or facilitators to intervention delivery and uptake - will be presented within each category by qualitative and quantitative data.

### Category 1: Implementation

### a. *Trial Enrolment*:

A total of 880 caregivers consented to participate in the trial. After consenting, 66 caregivers dropped from the study. Out of these 66 who dropped, 49 (74%) caregivers withdrew because they were unable or unwilling to provide stool samples, 14 (21%) chose not to continue with the study, and 3 (5%) caregivers did not continue because they moved out of the study area, could not be traced, and due to death of the infant.

### b. Intervention Reach and dose:

A total of 814 caregivers were successfully enrolled into the intervention, which translates to the intervention attaining a 93% reach. Out of all the caregivers who were enrolled into the intervention, 753 (93%) caregivers participated in the four household visits (Table 3).

**Table 3:** Number of caregivers who completed each of the intervention visits

Visits	Intervention group		Total N = 814	Two proportion Z test p-value (CI)
	Control (n = 390)	Intervention (n = 424)		
Visit 1 (23 weeks)	384 (98%)	421 (99%)	805 (99%)	0.26 (-0.02, 0.01)
Visit 2 (25 weeks)	371 (95%)	415 (98%)	786 (97%)	0.03 (-0.05, -0.00)
Visit 3 (29 weeks)	363 (93%)	409 (97%)	772 (95%)	0.03 (-0.06, -0.00)
Visit 4 (32 weeks)	353 (91%)	400 (94%)	753 (93%)	0.03 (-0.07, 0.00)

Of the 61 caregivers who did not complete the intervention visits, 36 (59%) dropped out because they moved out of the study area, 9 (15%) dropped out because of withdrawals (from the primary caregivers and their spouses), 9 (15%) could not be traced, 6 (10%) had travelled out of the study area, and 1 (2%) dropped out because of hospitalisation.

### c. Fidelity:

The first, second, third and fourth visits were to be conducted at 7, 21, 49 and 70 days after enrolment respectively. The visits, however, could be made within a window of ± seven days, to cater for caregivers who may not be available during the exact visit days (Table 2).

The results indicate that most of the visits by CHVs and the field staff involved in intervention activities were made within the recommended period. Out of the 805 completed first visits, approximately 78% (n = 628) were made within 14 days, with the rest being made after the 14 days. Of the 786 completed second visits, 81% (n = 633) were made within 14–28 days, approximately 17% (n = 137) were made after 28 days, and 2% (n = 16) were made before 13 days. Among the 772 caregivers who completed the third visits, more than half (51%, n = 392) were visited by CHVs within 42–54 days, and the rest were visited before (13%, n = 101), or after (36%, n = 279). A total of 753 fourth visits were completed. Approximately 66% (n = 494) of the fourth visit by the intervention field staff were made within 63–77 days, and 32% (n = 240) were made after 77 days.

Notably, visits were delayed or made after the recommended period because caregivers were way from home or had travelled. Debriefing sessions with CHVs and intervention field staff explored reasons for deviations from planned timings from the recommended schedule. Both CHVs and intervention field staff expressed difficulties in reaching caregivers, noting that caregivers were away from home during the planned visit period, as they had to attend to other errands (e.g. work). Often, CHVs and the intervention field staff made the visits at a later date when the caregivers were available.

### Category 2: Receipt and Mechanisms of Change

### a. Participants’ Engagement

During the fourth visit, caregivers (48/50) in correctly mentioned the items they received, their use, and the behavioural practices they had discussed with the intervention field staff and CHVs during previous visits.

“*The bowl, spoons, cup and table mat….one has to wash hands before serving and feeding the child…the child has to be fed from a clean environment…. after this, the feeding items are cleaned …and kept on a clean surface……”* (Primary caregiver, Western A Unit, Nyalenda A)

When presented with the motivational messaging items during the fourth visit, the caregivers (48/50) did not need to have the images on the calendar explained to them. They were rather able to explain the messages and further related the outcomes of their improved hygiene practices to their infant’s success and well-being. For example;

“*Proper hygiene has led to the child growing up and obtaining her degree”* (Primary caregiver, Central Unit, Nyalenda A)

There were few respondents (2/50) who could not remember the messages discussed or the items given in previous visits. For instance, a secondary caregiver did not know anything about the message that was given to the primary caregiver. When asked about the items, she said

*“I do not know where she kept them”* (Secondary caregiver, Western B1 unit, Nyalenda B).

### b. Participants’ Responses

With regards to the messages on the motivational calendars, all the caregivers specifically mentioned the hygienic practices, singling out the four main behaviours shown in the calendars. The caregivers interacted with the calendars by marking the days when their infants experienced diarrhoea and could tell when the children experienced diarrhoea, to the extent of being very specific on the dates; e.g.

‘…*Four days in November…*” (Primary caregiver, Central Unit, Nyalenda A)

Caregivers with no calendar markings (20/50) justified that their infants did not experience any diarrhoeal episodes. We also encountered two secondary caregivers who reported being aware of the study and participating in the intervention activities. For example, during the fourth visit, a male (secondary) caregiver reported that although the intervention field staff had delivered the intervention to the infant’s mother as the primary caregiver during the previous visits, he had been informed about the study and he participated by marking the calendar when his infant experienced diarrhoea.

Diarrhoea was seen as a normal occurrence at certain stages of an infant’s growth such as during teething, at the onset of weaning, and when infants began crawling.

*“It* [diarrhoea] *is because of teething….…he had a little diarrhoea, and I thought it was not sickness……He still has diarrhoea now because his upper teeth are coming out” (*Primary caregiver, Dago Unit, Nyalenda A*)*

### c. Acceptance: Utilisation and Behavioural Uptake of the Intervention

Intervention participants mentioned the benefits they experienced from using each of the intervention items. They specifically singled out benefits such as improved hygiene practices (e.g., separating infants’ items from the rest of the household items), a general reduction in the occurrence of diarrhoea, and that the behavioural practices had become habits. The next section presents findings on observed intervention items in the households, and the uptake of the specific food hygiene behaviours. Findings of the observed intervention items during the midline visit are summarised in Table 4, and the effect of the intervention on uptake of the food hygiene behaviours is summarised in Table 5.

**Table 4:** Summary of intervention items observed during the midline visit in intervention households

Variable	Frequency (%) (N = 387)
Handwashing container	373 (96%)
Motivational material	368 (95%)
Use of Safe Start feeding cup	224 (57.9%)
Use of Safe Start feeding bowl	88 (22.7%)
Use of Safe Start feeding spoon	154 (39.8%)
Use of Safe Start food storage container	18 (4.7%)

**Table 5:** Summary of behavioural practices during food preparation and feeding observations

Variable	Total	Control (%)	Intervention (%)	OR (CI)	P value*
	723	336 (46.5)	387 (53.5)		
**HANDWASHING**					
Handwashing observed before food preparation	445	193 (43.3%)	252 (56.7%)	1.38 (1.02–1.87)	0.03
Handwashing before feeding	279	133 (47.7%)	146 (52.3)	0.92 (0.68–1.25)	0.61
**HYGIENIC FEEDING**					
Heating food to a boil before serving	367	160 (43.6%)	207 (56.4%)	1.23 (0.94- 1.70)	0.12
*Feeding method* • Alternative methods (Hand feeding and feeding bottle) • Use of feeding utensil	54 645	39 (72.2) 273 (42.3)	15 (27.8) 372 (57.7)	3.54 (1.91- 6.56)	0.0001
**FOOD STORAGE**					
Food discarded	5	2 (40%)	3 (60%)		
Returned to cooking vessel	9	6 (66.7)	3 (33.3)		
Stored in covered container	318	1 (0.3)	317 (99.7)		
Stored in sealed container	391	327 (83.6)	64 (16.4)	0.00 (0.00 -0.004)	0.0001 ^β^ *

^β^A Comparison of storage in sealed containers and storage by covering in a container or cooking vessel using a lid or other covering

#### i. Handwashing with soap at Critical Times

A total of 723 caregivers (336 in the control group and 387 in the intervention group) participated in structured observations during food preparation. Out of these, approximately 62% (n = 445) of caregivers washed their hands before food preparation and only 39% (n = 279) of the caregivers washed their hands before feeding their infants. The provided handwashing containers were observed in almost all (96%; n = 373) of the intervention households during the midline food preparation observations that were conducted at 33 weeks (approximately 77 days after enrolment). During the intervention visits, caregivers reported that the handwashing containers made handwashing easy and regular for them and other household members, that the containers saved time (used during handwashing) compared to using basins, and that the dedicated location for the handwashing container and soap facilitated and made handwashing easier. All the caregivers reported that they had taken up handwashing at critical times (such as before feeding their infants and after toilet use), and that other household members had also adopted handwashing with soap.

‘…O*ther children have also made it a habit that they must first wash hands before eating*’ (Primary caregiver, Got Owak Unit, Nyalenda B)

Reported challenges of using the handwashing containers included lack of a dedicated space within the house, a general lack of water to refill the containers, and fears of the containers being stolen if kept outside the household. Due to these concerns, some households (3/50) kept the containers away and/or only used them at specific times/when there was need.

From the structured observations conducted during the midline food preparation observations, we found that caregivers in the intervention arm had 1.38 (95% CI: 1.02–1.87) times the odds of washing their hands before preparing infant food compared to caregivers in the control group (p = 0.035). However, we found no evidence of the intervention’s effect on caregivers’ handwashing practices before infant feeding (OR = 0.92; 95% CI 0.68–1.25; p = 0.6).

#### ii. Hygienic Feeding

During the food preparation observations, over 90% (n = 675) of the foods that were prepared from the 723 caregivers that were observed were ‘liquid’ foods, which included porridge (63%, n = 455), milk (25%, n = 180) and tea with milk (5%, n = 40). With regards to feeding the infants, a total of 700 caregivers were observed feeding their infants. Out of these, 92% (n = 646) of the caregivers used a feeding utensil (spoon, bowl or cup), 7% (n = 48) fed their infants using a feeding bottle, and less than 1% (n = 6) hand fed their infants. More than half (58%, n = 224) of the 387 intervention participants used the ‘Safe Start’ cup to feed their infants, 40% (n = 154) used the spoon, and 23% (n = 88) used the bowl during feeding (Table 4). During the intervention visits, caregivers (48/50) reported that infants enjoyed using the feeding items:

‘…T*he children recognise their feeding items* which *motivates them to feed.*’ (Primary caregiver, Dago unit, Nyalenda A)

There, however, were concerns with the items; for example, two caregivers noted that the spoon was not used because it was small, and two others reported that infants did not like using the cup. Discussions further revealed that there were other caregivers (fathers, nannies, neighbours, and older children) within the household or in the compound who took care of the infants (including feeding the infants), and that during feeding, the infants inserted several other objects in their mouths.

*“Sometimes the other children carry him…. sometimes they give him lollipop, biscuits….…. since he is over six months, everyone wants to feed him” (P*rimary caregiver, Western A unit, Nyalenda A)

In addition, two caregivers noted that they sometimes left the infants to crawl or move around even during feeding, and infants often touched other items or surfaces that were not clean.

*“After I place him down…he crawls and goes outside the house…. sometimes he puts dirty items in his mouth” (*Primary caregiver, Western A unit, Nyalenda A)

Results from the structured observations indicated improved hygienic feeding practices among caregivers in the intervention arm who had 3.5 (95% CI: 1.91–6.56) times the odds of using a feeding utensil compared to caregivers in the control group (p = 0.00).

#### ii. Hygienic Storage and Reheating

Half (n = 367) of the 723 foods prepared during the midline stage were heated to a boil before serving or feeding the infants, with more caregivers in the intervention group (56%, n = 207) heating food to a boil compared to caregivers in the control group (43%, n = 160). After feeding, over half (54%, n = 391) of all the 723 caregivers that were observed stored the left-over food in sealed containers, 44% (n = 318) covered (in a container or cooking vessel using a lid or other covering) the left-over food, and the rest (2%, n = 14) discarded the left-over food or returned the food to the cooking vessel. Only 16% (n = 64) of the 387 caregivers in the intervention arm stored leftover food in the provided sealed storage containers. All the caregivers in the intervention group reported that the food storage containers prevented dirt from food, they made storage of left-over food easier, and that the storage containers were very useful for packing infant food when caregivers and the infants were travelling.

Caregivers (7/50) reported that the storage containers were small and could only hold a small amount of food.

*“They are small…sometimes porridge does not fit in the containers...he is growing and the quantity of food [he consumes] also increases”* (Primary caregiver, Kilo 2 unit, Nyalenda B).

Additionally, four caregivers felt that the food storage containers did not keep food hot, which necessitated reheating of infant food. These caregivers explained that they often cooked in the morning and admitted that reheating food during the day was challenging since they did not always have fuel.

*“...I may have run out of fuel and I had cooked already, and yet I am required to reheat the food before feeding the child……..and there are others who do not use gas* [for cooking]*…they will not light a jiko* [charcoal burning stove] *to reheat the food. They will just feed the child without reheating” (Primary Caregiver, Got Owak, Nyalenda B)*

Others (4/50) noted that reheating was a time-consuming process, especially when the infants were hungry and needed to be fed immediately.

*“…. Reheating takes time…. many times I feed him the porridge without reheating…” (F*emale caregiver, Kanyakwar unit, Nyalenda A)

To overcome these challenges, caregivers made use of other storage items such as food flasks. Evidently, results from the food preparation observations indicated that majority (82%, n = 317) of the 387 caregivers in the intervention arm simply covered left over food in a container or cooking vessel using a lid or other covering; as opposed to storing food in the provided sealed containers. Thus, the intervention did not have an effect on caregivers’ hygienic food storage practices (OR 0.00; CI 0.00 -0.004; p = 0.0001).

#### Other Emergent Contextual Findings from Discussions with Caregivers

All the caregivers who were interviewed were involved in small-scale income generating activities or were informal workers around Kisumu. When they were out for work, they left the infants at home with secondary caregivers (partners, neighbours, nannies or older children). They also left the infants behind when attending social functions such as funerals or community/group meetings. When caregivers did not have a secondary caregiver, they took their infants to their workplaces or to social functions. Eight caregivers (out of 50), for example, had the intervention items delivered to them while at their business places such as small shops/grocery shop and at workplaces such as a restaurant, school and hospital. Caregivers were often not at home (hence the difficulty in tracing them), and/or they dedicated little time to some childcare activities. It is also possible that caregivers did not carry the intervention items with them when they travelled to their rural homes (perhaps only the storage containers).

## Discussion

The ‘Safe Start’ intervention reached 93% of eligible caregivers in the study area, and over 90% of recruited caregivers received and completed the intervention activities. With regards to fidelity, the intervention, on average, was delivered within the recommended time periods. We experienced a high engagement of the recipients with the intervention as reflected in the caregivers’ ability to recall the intervention items, and the use of the food hygiene items provided to the caregivers. Caregivers improved their infant food hygiene practices, specifically handwashing before food preparation, and feeding infants using separate feeding items. Contextual factors played an important role in both implementation and uptake of the intervention, especially the movement of caregivers within and outside the study area, and the unavailability of primary caregivers due to their involvement in income generating activities for household needs.

Over 90% of all caregivers who were recruited into the intervention completed the intervention visits. Nonetheless, we experienced a high movement of caregivers which resulted in losses to follow up during the intervention period. The high movement of residents is common in low income settlements where residents not only move between their rural homes and the low income settlements, but also within the settlements in search of income opportunities (Vijver et al., 2015; Knee et al., 2020). One of the strengths of the intervention was in the incorporation of CHVs in intervention delivery. CHVs are usually drawn from the community and have been effective in linking the community to the health system (Parvez et al., 2018, Oliver et al., 2015). In our case, we built on the existing system by using CHVs to deliver the intervention through their routine community visits while at the same time collecting vital statistics from the households during these visits.

During these visits, caregivers were encouraged to practice improved food hygiene. Results indicated that the intervention influenced the caregivers’ handwashing practice before food preparation, as has been observed in other food hygiene intervention studies in Africa (Chidziwisano et al., 2020; Geresomo et al., 2018). Possible reasons may be because of improved knowledge and the provision of the necessary hardware, in this case water storage containers and soap to facilitate hand washing (Chidziwisano et al., 2020; Dreibelbis et al., 2013; White et al., 2020; Chidziwisano et al., 2019). It is noted that in nutritional and child development programs, caregivers in low income countries may have challenges in adopting new messages and behaviours especially due to time constraints (Black et al., 2015), and as such, practices that are seemingly inconveniencing are less likely to be adopted (Sanghvi et al., 2017). We, however, noticed that caregivers generally did not wash hands before feeding their infants. It is possible that since caregivers fed the infants immediately after food preparation, they were not keen to wash their hands before feeding (assuming they had already washed their hands before food preparation).

Results also showed that the intervention influenced the caregivers’ usage of hygienic feeding items, including spoons, bowls and cups. Adoption of the feeding items was possible because the feeding items were familiar to caregivers and the infants (e.g. cups and bowls), they were attractive to the infants, and could easily be designated specifically for infant use. As such, the feeding items themselves motivated the infants during feeding. This may have been an advantage to caregivers especially because it would free some time spent on feeding the infants for other activities. Most of the foods that were prepared were liquid foods and the cup for example made feeding easier for the infants and the caregivers. These results are supported by literature that suggests that behaviour change approaches that are more impactful in improving complementary feeding are those that focus on generating positive emotions centred on the child’s well-being, and which are also convenient to the mother (Sanghvi et al., 2017). These results also suggest that strategies that promote easy and quick feeding of the infants would be easily adopted by caregivers since they encourage infant feeding and save time for caregivers.

Reheating and storage of infant food were less practiced by the caregivers. Lack of or inadequate reheating is a practice that has been noted among caregivers in other settings, which unfortunately, increases the chances of infant food contamination (Bick et al., 2020; Chidziwisano et al., 2020; Doza et al., 2018, Touré et al., 2011). In our setting, qualitative results suggested that caregivers were less likely to reheat food because of various reasons such as saving time spent in reheating, avoiding the additional task of cooling the food before feeding the infants, or the lack of fuel. Additionally, as highlighted from formative results, caregivers prepared infant food when preparing food for the family, and this food was stored in food flasks (Mumma et al., 2020). These economic and contextual factors explain the reason for the low uptake of the food storage containers. Caregivers preferred to prepare infant food in large quantities and store it in food flasks to avoid additional tasks and fuel costs, and thus the food storage containers were used to store other infant food that did not require reheating or that they were used to store infant food when the caregivers travelled.

With regards to context, we noted that infants had multiple caregivers within the household, who ranged from members within and without the household (Mumma et al., 2020). Since primary caregivers left in search of income opportunities, the infants were often left behind with secondary caregivers, who may not have been keen to practice the food hygiene behaviours especially when infants were fed by multiple individuals. Secondary caregivers may be key targets for such programs and their involvement may have significant outcomes in overall infant health. These findings point to the importance of targeting secondary caregivers within the household who spend a significant amount of time with infants in future studies (Mumma et al., 2020).

Our study has various limitations. First, observations were made during food preparation and feeding, and for a short period. There is a risk of reactivity as observations tend to influence participant behaviours (Ram et al., 2010) especially in an open trial, and the short observation period may not reflect the actual day-to-day practices. To address this limitation, we complemented the observation data with qualitative findings from the caregivers during the intervention period, which provided further contextual explanation to the quantitative findings from the structured observations. In this manuscript, we did not account for and evaluate the role of covariates on the behavioural outcomes reported. These will be included in the forthcoming outcome paper. Finally, the intervention lacked an adequate monitoring plan to validate if and when the visits had actually been done, and the information that had been passed to caregivers.

## Conclusion

The Safe Start trial was conducted in a low-income peri-urban settlement in Kenya to evaluate an intervention to improve infant health through food hygiene practices among caregivers. This process evaluation has documented the implementation of the trial in its context and has explored associations between exposure to the intervention and its adoption. The intervention had high reach and with over 90% of the target caregivers receiving all intervention components. We succeeded in improving some targeted food hygiene behaviours, particularly handwashing with soap before food preparation and hygienic feeding of infants. Adoption of the intervention was influenced by contextual factors including high rates of mobility within and without the settlements and household level factors such as low rates of reheating of food due to lack of time and resources to purchase cooking fuel. Our study provides valuable lessons for the design, implementation and evaluation of similar interventions in such settings, for example, it is necessary to explore the possibilities of promoting the use of insulated food storage containers especially when reheating is challenging. Low rates of handwashing with soap after cooking and before feeding may imply that handwashing messages should be promoted as independent events and not dependent of each other, for example, handwashing messages should be clear that handwashing before feeding is necessary, even if handwashing had already been done before. Finally, this study confirms the value of embedding process evaluations in similar trials/studies, since these evaluations help interpreting the outcomes of the studies/trials, and identifying successful aspects of an intervention which informs future programming and studies.

## Data Availability

Not applicable.
